# miR-1291 Functions as a Potential Serum Biomarker for Bullous Pemphigoid

**DOI:** 10.1155/2020/9505312

**Published:** 2020-01-11

**Authors:** Li Qiu, Liming Zhang, Ruiqun Qi, Xinghua Gao, Hongduo Chen, Ting Xiao

**Affiliations:** Department of Dermatology, The First Hospital of China Medical University, National Health Commission Key Laboratory of Immunodermatology, Key Laboratory of Immunodermatology of Ministry of Education, Shenyang, Liaoning, China

## Abstract

**Background:**

Bullous pemphigoid (BP) is a common T helper 2- (Th2-) dominated autoimmune blistering skin disease with significant mortality. MicroRNAs (miRNAs), which are endogenous noncoding RNA molecules, have been reported to be potential biomarkers for some autoimmune diseases; however, to date, there exist no reports on serum expression profiles of miRNAs in BP patients.

**Methods:**

A RNA quantitative PCR- (qPCR-) based array was conducted on sera from 20 active BP patients and 20 healthy controls for screening of miRNAs. Significantly dysregulated miRNAs were validated with use of qPCR as performed on sera samples of 45 active BP patients and 60 healthy controls. Serum CCL17, anti-BP180, and anti-BP230 levels were measured with use of ELISA.

**Results:**

Relative baseline expression levels of serum miR-1291 were significantly upregulated in the 45 BP patients as compared with the 60 healthy controls (*P* < 0.001) and significantly decreased in the disease control stage (*n* = 13, *P* = 0.006). In addition, these baseline miR-1291 levels showed a significant positive correlation with the baseline levels of serum CCL17 (*P* < 0.001) and anti-BP180 (*n* = 38, *P* = 0.024). Like that observed for miR-1291, baseline levels of serum CCL17 were also significantly elevated in the 45 BP patients compared with the 60 healthy controls (*P* < 0.001) and significantly decreased in the disease control stage (*n* = 13, *P* = 0.002). However, for anti-BP180, baseline serum levels were significantly elevated in only 38 of the 45 BP patients and significantly decreased in the disease control stage (*n* = 10, *P* = 0.004).

**Conclusions:**

Relative expression levels of serum miR-1291 can reflect disease activity of BP. miR-1291 may function as an important new serum biomarker for BP.

## 1. Introduction

Bullous pemphigoid (BP) is a T helper 2- (Th2-) dominated autoimmune subepidermal blistering skin disease resulting from circulating autoantibodies directed against structural components of hemidesmosomes. These autoantibodies mainly consist of bullous pemphigoid antigen 2 (BPAG2, BP180) and bullous pemphigoid antigen 1 (BP230, BPAG1) [1]. Serum levels of anti-BP180 antibody have been reported to be elevated in 72-93% of BP patients and are correlated with disease activity of BP, while anti-BP230 antibody levels are detected in only 57-63% of BP patients and do not reflect BP disease activity [2]. Th2-type inflammation plays an important role in the pathogenesis of BP, and CCL17 is a well-recognized Th2 chemokine. Serum CCL17 levels have been reported to be significantly elevated in active BP patients and reflect disease activity of this condition [3–5]. In addition to BP, serum CCL17 levels are also significantly elevated in patients with other Th2-dominated diseases including atopic dermatitis (AD) and asthma and can also serve as an index of disease activity for these two conditions [6, 7]. In spite of these relationships, use of anti-BP180 and CCL17 as serum biomarkers for BP is relatively restricted due to their limited sensitivity and specificity. Accordingly, new types of biomarkers are needed for use in the diagnosis of BP.

MicroRNAs (miRNAs) are endogenous noncoding RNAs that play important roles in gene transcription and expression. miRNAs regulate the expression of at least half of the human transcriptome by either repressing the translation of or degrading multiple-target mRNAs. This silencing mechanism can be determined by assessing the extent of base pairings between the miRNA and the target mRNA, when the mRNA binds to the complimentary target sites located in the 3′ untranslated region of the target mRNA [8]. miRNAs are secreted by cells through exosomes and extracellular vesicles, and these secreted miRNAs can remain stable in body fluids with a half-life of ≥5 days [9]. Recently, miRNAs have been shown to be key factors in autoimmunity [10, 11] and, in this way, can be used as diagnostic biomarkers for some autoimmune diseases including pemphigus, systemic lupus erythematosus, and systemic sclerosis [12–14]. However, the expression profiles of miRNAs, which could serve as biomarkers in sera of BP patients, have not been reported in the literature. The aim of this study was to investigate differentially expressed miRNAs in sera of BP patients and assess their roles as serum biomarkers for BP by comparing levels of these differentially expressed miRNAs with those of serum CCL17 and anti-BP180 levels.

## 2. Materials and Methods

### 2.1. Participants

A total of 65 ethnic Han Chinese active BP inpatients were assessed over the period from June 2016 to June 2018. The diagnostic criteria for BP were in accordance with the established European guidelines [15]. BP patients treated with systemic corticosteroids or immunosuppressants, not in the active stage of the disease, showing malignant tumors and/or other autoimmune diseases were excluded from the study. Eighty healthy ethnic Han Chinese served as controls. The study was approved by the ethics committee of the First Hospital of China Medical University (2018-307-3).

### 2.2. Clinical Data

Patient-level data were obtained from the electronic medical records. The records included age, sex, ethnicity, disease duration, previous history, IIF titer, and treatment regimens of BP.

### 2.3. Assessment of BP Disease Activity

Baseline was defined as the time at which a physician started treatment for BP. Disease control was defined as the time at which formation of new lesions or pruritic symptoms ceased and established lesions began to heal [15].

### 2.4. Quantitative Polymerase Chain Reaction- (qPCR-) Based Array

For screening, a qPCR-based array was performed as previously described [16]. Briefly, total RNA was isolated from serum samples of the 20 active BP patients and 20 healthy controls with a miRNeasy Serum/Plasma Kit (Qiagen, Hilden, Germany). The yield of total RNA was 7-15 ng/*μ*L. The cDNA was obtained from 3 *μ*L of RNA with use of the TaqMan® MicroRNA Reverse Transcription Kit (Applied Biosystems, Beverly, USA). The TaqMan® PreAmp Kit (Applied Biosystems) was used for preamplifying with 2.5 *μ*L of cDNA product per specimen. The qPCR-based array analysis was performed with use of the TaqMan Low Density Array Human MicroRNA Panel (CapitalBio, Beijing, China). A total of 768 known miRNAs were quantified with use of the TaqMan® Human MicroRNA Array A and B (Applied Biosystems). Raw cycle threshold (Ct) values were calculated by SDS 2.4 and RQ manager 1.2 software (Applied Biosystems). A Ct value of ≥35.0 was used as a cut-off.

### 2.5. qPCR Validation Study

C. elegans miR-39 at 1.6 × 10^8^ copies/*μ*L (Qiagen) was added to the sera prior to RNA isolation. Total RNA was isolated from serum samples of the 45 active BP patients and 60 healthy controls. Extracted RNA was diluted to a uniform concentration of 2 ng/*μ*L, and the reverse transcription reaction was performed with use of a Reverse Transcription Kit (Applied Biosystems). miRNAs were quantified with use of TaqMan™ MicroRNA assays (Applied Biosystems). The qPCR was performed with a 7900HT Fast Real-Time PCR System (Applied Biosystems). Each qPCR test was performed in duplicate. Ct values were obtained with use of SDS 2.4 and RQ manager 1.2 software (Applied Biosystems). The Ct values (mean ± SD) of C. elegans miR-39 in the BP patients and the healthy controls in this study were 32.51 ± 1.31 and 32.51 ± 1.10, respectively. There were no statistically significant difference (*P* = 0.45) and only a very small variation between the two groups. We chose C. elegans miR-39 to normalize the relative expression levels of miRNAs [16, 17].

### 2.6. Enzyme-Linked Immunosorbent Assay (ELISA)

Serum levels of CCL17 were determined with use of ELISA kits (R&D Systems, USA). The minimal detectable concentration of CCL17 was 7 pg/mL. Serum levels of anti-BP180 and anti-BP230 were also determined using ELISA kits (MBL Systems, Nagoya, Japan). The normal ranges of anti-BP180 and anti-BP230 were both 0-9 U/mL.

### 2.7. Statistical Methods

For analysis of qPCR-based arrays, data were standardized as based on the global normalization method. The *Δ*Ct value for each individual miRNA was equal to the Ct value, as obtained by subtracting the mean Ct value from all detected miRNAs in each specimen [16]. qPCR-based array data were analyzed using software R (version 3.2.3). Differentially expressed miRNAs were defined as satisfying a criterion of both a minimum of a twofold change in 2^∣-*ΔΔ*Ct∣^ and a *P* value < 0.05 between BP patients and healthy controls. Relative expression levels of serum miRNAs were normalized to cel-miR-39 (*Δ*Ct) and presented as 2^-*Δ*Ct^ in the qPCR validation study.

Results for nonnormally distributed continuous variables were presented as medians (and interquartile ranges (IQR)), and Mann-Whitney *U* tests were used to compare differences between BP patients and healthy controls. Data from qualitative variables were analyzed with use of Fisher's exact test. Wilcoxon signed-rank tests were performed for paired data. Receiver operating characteristic (ROC) curves and areas under the curve (AUC) were performed to evaluate the diagnostic performance of miRNAs and CCL17. The maximal Youden's index was calculated with the ROC curve, with Youden′s index = sensitivity + specificity − 1. The optimal cut-off value was set as the threshold with the maximal Youden's index [16]. Correlations between significantly dysregulated miRNAs and significantly elevated levels of CCL17 and anti-BP180 were analyzed with use of Spearman's rank correlation coefficient. *P* values < 0.05 were required for results to be considered statistically significant. Statistical analysis was performed with use of the GraphPad Prism 7.0 program.

### 2.8. Bioinformatics Methods

Targeted genes of significantly dysregulated miRNAs as predicted by computer-aided algorithms were obtained from the TargetScan miRNA database, miRDB, and TarBase. As miRNA databases predict targets based on sequences alone, there is some element of chance involved. To avoid this possibility, we considered only those potential targets reported in the literature as being related to BP. The KEGG pathway database [18] and PathCards database [19] were used to assess pathways of the targeted genes.

## 3. Results

### 3.1. Characteristics of BP Patients and Healthy Controls

The demographic, clinical, and laboratory data of the participants in the qPCR-based array analysis and the qPCR validation study are presented in Table 1. The initial therapeutic regimens of the 45 BP patients in the qPCR validation study are listed in Table 2.

### 3.2. Significantly Dysregulated miRNAs in the qPCR-Based Array Analysis

**Figure 1:**
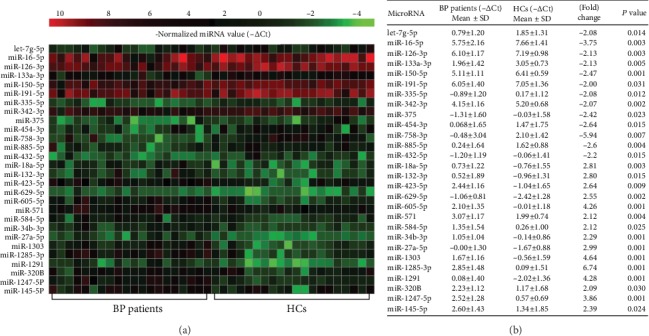
Serum miRNAs differentially expressed within 20 BP patients as compared with the 20 healthy controls (HCs). (a) Heat map illustrates significantly dysregulated miRNAs using qPCR-based array. The vertical axis is constructed using the -*Δ*Ct values of all 28 significantly upregulated or downregulated miRNAs (*P* < 0.05). The horizontal axis represents the 20 patients and the 20 HCs. (b) The mean -*Δ*Ct values, fold change, and *P* values of the 13 significantly downregulated miRNA (with positive fold change) and the 15 significantly upregulated miRNAs (with negative fold change) in BP patients (*P* < 0.05).

Totally, 327 miRNAs were detected, of which 15 miRNAs were significantly upregulated and 13 downregulated in the sera of the 20 active BP patients as compared with those obtained in the 20 healthy controls (Figure 1).

### 3.3. Significantly Dysregulated miRNAs in the qPCR Validation Study

**Figure 2:**
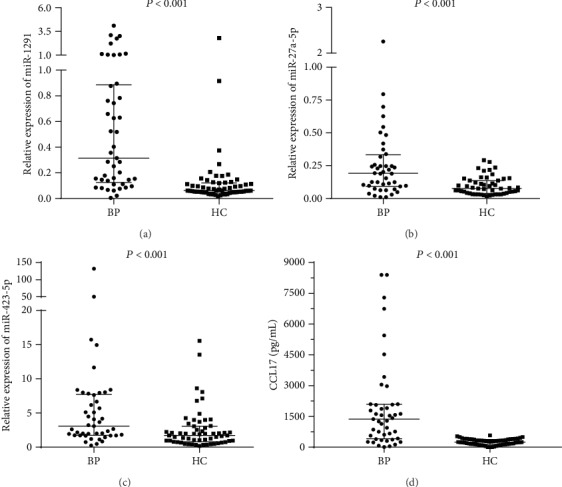
Relative expression levels of serum (a) miR-1291, (b) miR-27a-5p, (c) miR-423-5p, and (d) serum CCL17 levels of the 45 patients and the 60 healthy controls are plotted. The median (and interquartile range) values are shown. BP: bullous pemphigoid; HC: healthy control.

Among the 28 significantly dysregulated miRNAs in the qPCR-based array study, relative expression levels (median and IQR) were all significantly upregulated in baseline sera of the 45 active BP patients in comparison with the 60 healthy controls for serum miR-1291 (0.31 (0.13-0.89) vs. 0.06 (0.05-0.12), *P* < 0.001, Figure 2(a)), miR-27a-5p (0.19 (0.09-0.33) vs. 0.08 (0.04-0.14), *P* < 0.001, Figure 2(b)), and miR-423-5p (3.09 (1.74-7.74) vs. 1.73 (0.79-3.08), *P* < 0.001, Figure 2(c)).

### 3.4. Baseline Serum Levels of CCL17, Anti-BP180, and Anti-BP230 in the 45 Active BP Patients

Baseline levels (median and IQR) of serum CCL17 in the 45 active BP patients were significantly increased as compared with those of the 60 healthy controls (1368 (410-2088) pg/mL vs. 248.5 (143.4-376.7) pg/mL, *P* < 0.001, Figure 2(d)). Of the 45 active BP patients, 38 (84.44%) had serum levels of anti‐BP180 > 9 U/mL (71.88 (41.6-123.6) U/mL) and 25 (55.56%) had serum levels of anti‐BP230 > 9 U/mL (82.48 (50.51-106.6) U/mL).

### 3.5. Relative Expression Levels of Serum miR-1291, CCL17, Anti-BP180, and Anti-BP230 Are Significantly Decreased in the Disease Control Stage

**Figure 3:**
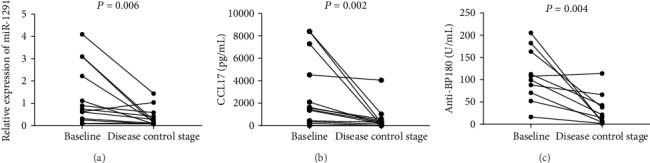
Relative expression levels of serum miR-1291 and levels of serum CCL17 and anti-BP180 in the active stage (baseline) and disease control stage of the 13 BP patients. As compared with baseline values, significant reductions in the disease control stage were obtained for (a) relative expression levels of serum miR-1291 (*P* = 0.006), (b) serum CCL17 levels (*P* = 0.002), and (c) serum anti-BP180 levels (*n* = 10, *P* = 0.004).

An additional sample of serum was obtained from the 13 BP patients in the disease control stage. Characteristics of these 13 patients are contained in Table 3. Elevated baseline serum levels were obtained in 10/13 BP patients for anti-BP180 and 9/13 for anti-BP230. In comparison with their baseline levels, relative levels (median and IQR) of serum miR-1291 expression in the 13 BP patients were significantly decreased in the disease control stage (0.74 (0.28-2.66) vs. 0.19 (0.08-0.50), *P* = 0.006, Figure 3(a)). In contrast, no significant reductions were observed in the disease control stage for relative expression levels of miR-27a-5p (0.34 (0.20-0.50) vs. 0.26 (0.11-0.54), *P* = 0.470) or miR-423-5p (4.63 (3.11-6.93) vs. 3.123 (1.55-4.3), *P* = 0.104). Accordingly, only miR-1291 appears to provide a reflection of BP disease activity. In addition, as compared with the baseline values, levels of serum CCL17 (1557 (387-5906) pg/mL vs. 246.3 (86.4-514.2) pg/mL, *n* = 13, *P* = 0.002, Figure 3(b)), anti-BP180 (103.5 (66.08-168.2) U/mL vs. 18.09 (4.80-48.58) U/mL, *n* = 10, *P* = 0.004, Figure 3(c)), and anti-BP230 (80.87 (27.32-102.9) U/mL vs. 21.61 (2.65-59.63) U/mL, *n* = 9, *P* = 0.008) were also all significantly decreased in the disease control stage.

### 3.6. Areas under the Curve (AUC), Sensitivity, and Specificity of miR-1291 and CCL17

**Figure 4:**
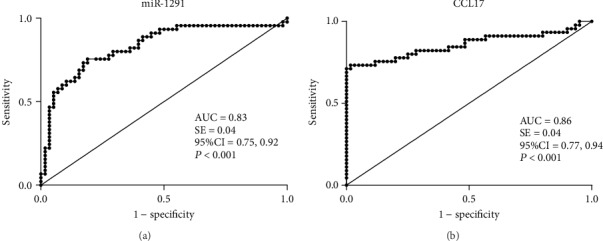
Receiver operating characteristic (ROC) curves and area under the curve (AUC) values of (a) miR-1291 and (b) CCL17 for the 45 BP patients and 60 healthy controls. An AUC value equal to 1.0 indicates that the parameter shows a perfect discrimination between BP patients and healthy controls. An AUC equal to 0.5 indicates purely random results.

The AUC value for miR-1291 was 0.83 (95% CI, 0.75-0.92, *P* < 0.001, Figure 4(a)). The sensitivity and specificity of miR-1291 for BP (2^-*Δ*Ct^ at cut-off level of >0.13) were 75.56% and 81.03%, respectively. The AUC value for CCL17 was 0.86 (95% CI, 0.77-0.94, *P* < 0.001, Figure 4(b)). The sensitivity and specificity of CCL17 for BP (at cut-off level of >549.0 pg/mL) were 73.33% and 98.33%, respectively.

### 3.7. Correlations between miR-1291 and CCL17 and Anti-BP180

**Figure 5:**
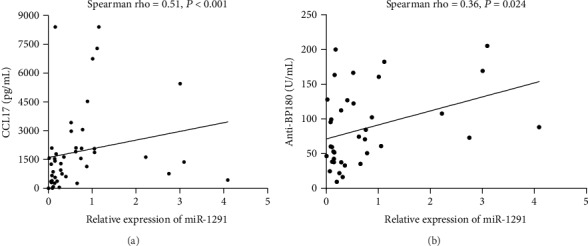
Relative expression of serum miR-1291 levels (*n* = 45) showed a statistically significant positive correlation with serum levels of (a) CCL17 (*n* = 45) and (b) anti-BP180 (*n* = 38) in BP patients.

Relative levels of miR-1291 expression showed a statistically significant positive correlation with serum CCL17 levels (Spearman rho = 0.51, *P* < 0.001, Figure 5(a)) and anti-BP180 (Spearman rho = 0.36, *P* = 0.024, Figure 5(b)). No statistically significant correlations were obtained between miR-1291 and anti-BP230.

### 3.8. Relative Expression of miR-1291 Levels in Different Subgroups of BP Patients

The 45 active BP patients were divided into different subgroups by sex, with/without hypertension, with/without cerebrovascular disease, with/without mucosal involvement, serum CCL17 levels > or ≤549.0 pg/mL, positive/negative anti-BP180, and positive/negative anti-BP230. Relative expression of serum miR-1291 levels (median and IQR) was significantly upregulated in the 33 patients with CCL17 > 549.0 pg/mL as compared with the 12 patients showing CCL17 levels of ≤549.0 pg/mL (0.52 (0.16-1.03) vs. 0.13 (0.08-0.24), *P* = 0.009). No other statistically significant differences in relative expression of serum miR-1291 levels were obtained among the other subgroups (Table 4). Of the 34 BP patients with serum levels of miR‐1291 expression > 0.13 (cut-off value), 28 (82.35%) had a serum CCL17 level > 549 pg/mL, while in the 11 BP patients with serum levels of miR‐1291 expression ≤ 0.13, 5 (45.45%) had serum CCL17 level > 549 pg/mL (*P* = 0.016). The 45 BP patients were divided into two groups according to the median of miR-1291 (0.31), and then the number of BP patients whose serum CCL17 levels > 549.0 pg/mL or anti‐BP180 > 60 U/mL was compared. Compared with the patient group with serum miR‐1291 level ≤ 0.31, the percent values of patients with CCL17 levels > 549.0 pg/mL or anti‐BP180 > 60 U/mL were both significantly higher in the patient group with serum miR‐1291 level > 0.31 (Table 5).

### 3.9. BP-Associated Targeted Genes of miR-1291

We found 1575, 469, and 55 predicted targeted genes of miR-1291 in the TargetScan miRNA database, miRDB, and TarBase, respectively. Based on the criteria described in the methods, signal transducer and activator of transcription 6 (STAT6) and interleukin-13 (IL-13) were selected as potential BP-associated targeted genes of miR-1291 in the TargetScan miRNA database, while IL-13 was selected as a potential BP-associated targeted gene of miR-1291 with miRDB [2, 20, 21]. No BP-associated targeted genes were found in the TarBase. Therefore, we chose STAT6 and IL-13 as possible BP-associated target genes of miR-1291. According to the KEGG and the PathCards database, the signal pathways associated with STAT6 and IL-13 are listed in Table 6. We found that the Janus kinase and signal transducer and activator of transcription (JAK-STAT) signaling pathway and Th2 cell differentiation pathway were shared signal pathways in the KEGG and the PathCards database.

## 4. Discussion

To the best of our knowledge, this study represents the first report on serum expression profiles of miRNAs in BP patients. Our results demonstrate that the relative expression of miR-1291 was significantly upregulated in the sera of active BP patients and significantly decreased after effective treatment. These miR-1291 responses were associated with a 75.56% sensitivity and 81.03% specificity. Such results suggest that miR-1291 can reflect disease activity in BP patients as well as their responses to treatment.

Significantly elevated serum levels of the Th2 chemokine, CCL17, in active BP patients have been reported previously in the literature [3–5]. In this study, we show that the relative expression levels of serum miR-1291 were significantly upregulated in the 33 active BP patients with CCL17 > 549.0 pg/mL as compared with the 12 active BP patients with CCL17 ≤ 549.0 pg/mL. We also found that relative baseline expression levels of serum miR-1291 showed a significant positive correlation with baseline levels of serum CCL17 and anti-BP180 in active BP patients. Therefore, like CCL17 and anti-BP180, miR-1291 may also prove to be a good, if not better, marker for assessing disease activity of BP. The percent values of patients with CCL17 levels > 549.0 pg/mL were significantly higher in the patients with serum miR‐1291 level > 0.13 than in the patients with serum miR‐1291 level ≤ 0.13. Compared with the patients with serum miR‐1291 level ≤ 0.31, the percent values of patients with CCL17 levels > 549.0 pg/mL or anti‐BP180 > 60 U/mLwere both significantly higher in the patients with serum miR‐1291 level > 0.31. Therefore, we think the high variability of serum miR-1291 levels in the BP patients reflects the various disease activity of BP.

In this study, STAT6 and IL-13 were chosen as potential BP-associated target genes of miR-1291. The expression of STAT6 has been reported to be significantly elevated in BP skin lesions, but not in BP perilesional skin and normal skin [20]. Dysfunction in regulatory T cells (Tregs) triggers STAT6-mediated activation of autoreactive CD4+ Th2 cells and follicular helper T cells, resulting in the production of autoantibodies to BP antigens [22]. With regard to IL-13, it has been reported that eosinophils are able to secrete IL-13 which promotes Th2 polarization [23], and these IL-13-producing cells are often observed in BP skin lesions. IL-13 contributes to the activation of type 2 macrophages, leading to a positive feedback for the induction of Th2 cells, and maintains the Th2-polarized immunological microenvironment in BP [21]. Given this background information, it was considered important to conduct further experiments to verify the regulatory relationships of these two target genes. We found that STAT6 and IL-13 are involved in the JAK-STAT signaling pathway and Th2 cell differentiation pathway. The JAK-STAT pathway is essential for normal functioning of the immune system. Interestingly, JAK-STAT signaling is dysregulated in BP, which may be related to activation of distinct cytokines and neutrophilic and/or eosinophilic infiltration [20]. The signature cytokines of Th2 cells are IL-4 and IL-13. IL-4 can promote the differentiation of Th2 cells. These Th2 cells can then express the chemokine receptors, CCR4 and STAT6, which serve as the major signaling pathway of IL-4-mediated Th2 differentiation. For IL-13, it has been reported that STAT6 induces GATA-3 expression and GATA-3 binds to the promoters of IL-13 and the enhancer of IL-4, thereby promoting their transcription [24].

There were some limitations in our study. Bullous Pemphigoid Disease Area Index (BPDAI) scoring was not performed in all patients. As many patients were from diverse locations, sera collected in the disease control stage were not obtained in all patients but only in some patients who were followed up regularly. As BP occurs mainly in the elderly, we were unable to recruit enough age-matched healthy controls for this study. In addition, no investigation was performed regarding *in vivo* functions of miR-1291 and the genes targeted by miR-1291. It is not clear what kind of cells or tissues release miR-1291 into serum, and there is still a long way to go between predicting the target genes and understanding the pathogenesis. This is an exploratory study to find the dysregulated microRNAs in sera of BP, making a preliminary discussion of the putative target genes, and give some hints to other researchers. Future studies of target genes and related pathways will be carried out in immune-related cells and lesional tissues of BP patients. While remaining aware of these limitations, we feel that the data presented provide important new information on initial investigations into the expression of miRNAs within the serum of BP patients.

## 5. Conclusions

Relative expression levels of serum miR-1291 reflect disease activity and treatment responses within BP patients. miR-1291 may function as a serum biomarker for BP.

## Figures and Tables

**Table 1:** Characteristics of the BP patients and the healthy controls in the qPCR-based array and qPCR validation studies.

	qPCR-based array study		qPCR validation study
	BP patients (*n* = 20)	HCs (*n* = 20)	BP patients (*n* = 45)	HCs (*n* = 60)
Age (years) median (IQR)	72.5 (60.75-81.75)	38.5 (30-54)	73 (61-82)	48.5 (42-55)
Sex (female/male)	12/8	16/4	20/25	26/34
Disease duration (months) median (IQR)	2 (0.62-3.5)	NA	2 (1-3.5)	NA
Previous history, *n* (%)	7 (35)	0	17 (37.78)	0
Hypertension, *n*	2	0	14	0
Cerebrovascular disease, *n*	7	0	9	0
Mucosal involvement, *n* (%)	2 (10)	0	10 (22.22)	0
Positive IIF, *n* (%)	16 (80)	NA	36 (80)	NA
Anti-BP180 (U/mL)				
>9, *n* (%)	16 (80)	NA	38 (84.44)	NA
Anti-BP230 (U/mL)				
>9, *n* (%)	NA	NA	25 (55.56)	NA
CCL17 (pg/mL) median (IQR)	NA	NA	1368 (410-2088)	248.5 (143.4-376.7)
miR-1291 (2^-*Δ*Ct^), median (IQR)	1.01 (0.33-2.03)	0.25 (0.07-0.51)	0.31 (0.13-0.89)	0.06 (0.05-0.12)

BP: bullous pemphigoid; qPCR: quantitative polymerase chain reaction; HCs: healthy controls; IQR: interquartile ranges; NA: not available.

**Table 2:** Initial therapeutic regimens of the 45 BP patients.

Therapeutic regimens	*N*
Systemic prednisone (or equivalent) (mg/kg/day)	39
<0.5	6
0.5-0.75	19
>0.75	14
Oral nicotinamide and minocycline and topical halometasone	6

**Table 3:** Characteristics of the 13 BP patients whose serum samples were collected in both the disease active and control stages.

	BP patients (*n* = 13)
Age (years) median (IQR)	57 (51-71.5)
Sex (female/male)	7/6
Female age (years) median (IQR)	56 (46-62)
Male age (years) median (IQR)	71.5 (51.5-79.25)
Disease duration (months) median (IQR)	2 (1-3.5)
Previous history, *n* (%)	5 (38.5)
Hypertension, *n* (%)	4 (30.8)
Cerebrovascular disease, *n* (%)	2 (15.4)
miR-1291 (2^-*Δ*Ct^), median (IQR), *n* = 13	
Baseline	0.74 (0.28-2.66)
Disease control stage	0.19 (0.08-0.50)
CCL17 (pg/mL) median (IQR), *n* = 13	
Baseline	1557 (387-5906)
Disease control stage	246.3 (86.4-514.2)
Anti‐BP180 > 9 U/mL, median (IQR), *n* = 10	
Baseline	103.5 (66.08-168.2)
Disease control stage	18.09 (4.80-48.58)
Anti‐BP230 > 9 U/mL, median (IQR), *n* = 9	
Baseline	80.87 (27.32-102.9)
Disease control stage	21.61 (2.65-59.63)

**Table 4:** Relative expression levels of serum miR-1291 within the different subgroups of bullous pemphigoid (BP) patients.

Subgroups	*n* = 45	miR-1291 (2^-*Δ*Ct^), median (IQR)	*P* value
Sex			
Female	20	0.52 (0.15-1.09)	0.093
Male	25	0.25 (0.09-0.71)	
Hypertension			
With	14	0.30 (0.12-0.55)	0.520
Without	31	0.31 (0.11-1.01)	
Cerebrovascular disease			
With	11	0.18 (0.08-0.63)	0.255
Without	34	0.34 (0.15-1.02)	
Mucosal involvement			
With	10	0.45 (0.15-1.02)	0.677
Without	35	0.31 (0.11-0.88)	
CCL17			
≤549.0 pg/mL	12	0.13 (0.08-0.24)	**0.009**
>549.0 pg/mL	33	0.52 (0.16-1.03)	
Anti-BP180			
0-9 U/mL	7	0.63 (0.08-1.05)	0.915
>9 U/mL	38	0.30 (0.14-0.81)	
Anti-BP230			
0-9 U/mL	20	0.23 (0.09-0.70)	0.113
>9 U/mL	25	0.63 (0.15-1.09)	

**Table 5:** Comparisons of percent values of patients with CCL17 levels > 549.0 pg/mL or anti‐BP180 > 60 U/mL in the two groups of patients with different miR-1291 levels.

Serum miR-1291	*n*	CCL17 > 549 pg/mL*n* (%)	Anti‐BP180 > 60 U/mL, *n* (%)
>0.31	23	22 (95.65)	15 (65.22)
≤0.31	22	12 (54.55)	7 (31.82)
*P*		0.001	0.025

**Table 6:** Signal pathways including STAT6 and IL-13 from the KEGG and the PathCards database.

Database	Signal pathway
KEGG	JAK-STAT signaling pathway
	Th2 cell differentiation pathway
	Inflammatory bowel disease

PathCards	JAK-STAT signaling pathway
	Th2 differentiation pathway
	Th17 cell differentiation
	ERK signaling
	Interleukin-4 and interleukin-13 signaling
	Cytokine signaling in immune system
	Akt signaling
	TGF-beta pathway

STAT6: signal transducer and activator of transcription 6; IL-13: interleukin-13; JAK-STAT: Janus kinase and signal transducer and activator of transcription; ERK: extracellular signal-regulated kinase; TGF-beta: transforming growth factor-beta.

## Data Availability

The data used to support the findings of this study are available from the corresponding author upon request.
